# Machine Learning in Antibacterial Drug Design

**DOI:** 10.3389/fphar.2022.864412

**Published:** 2022-05-03

**Authors:** Marko Jukič, Urban Bren

**Affiliations:** ^1^ Laboratory of Physical Chemistry and Chemical Thermodynamics, Faculty of Chemistry and Chemical Engineering, University of Maribor, Maribor, Slovenia; ^2^ Faculty of Mathematics, Natural Sciences and Information Technologies, University of Primorska, Koper, Slovenia

**Keywords:** artificial intelligence, machine learning, computer-aided drug design (CADD), infectious diseases, antibacterial drug design, antibacterial, antibacterial target discovery, antibacterial drug resistance

## Abstract

Advances in computer hardware and the availability of high-performance supercomputing platforms and parallel computing, along with artificial intelligence methods are successfully complementing traditional approaches in medicinal chemistry. In particular, machine learning is gaining importance with the growth of the available data collections. One of the critical areas where this methodology can be successfully applied is in the development of new antibacterial agents. The latter is essential because of the high attrition rates in new drug discovery, both in industry and in academic research programs. Scientific involvement in this area is even more urgent as antibacterial drug resistance becomes a public health concern worldwide and pushes us increasingly into the post-antibiotic era. In this review, we focus on the latest machine learning approaches used in the discovery of new antibacterial agents and targets, covering both small molecules and antibacterial peptides. For the benefit of the reader, we summarize all applied machine learning approaches and available databases useful for the design of new antibacterial agents and address the current shortcomings.

## Introduction

**FIGURE 1:**
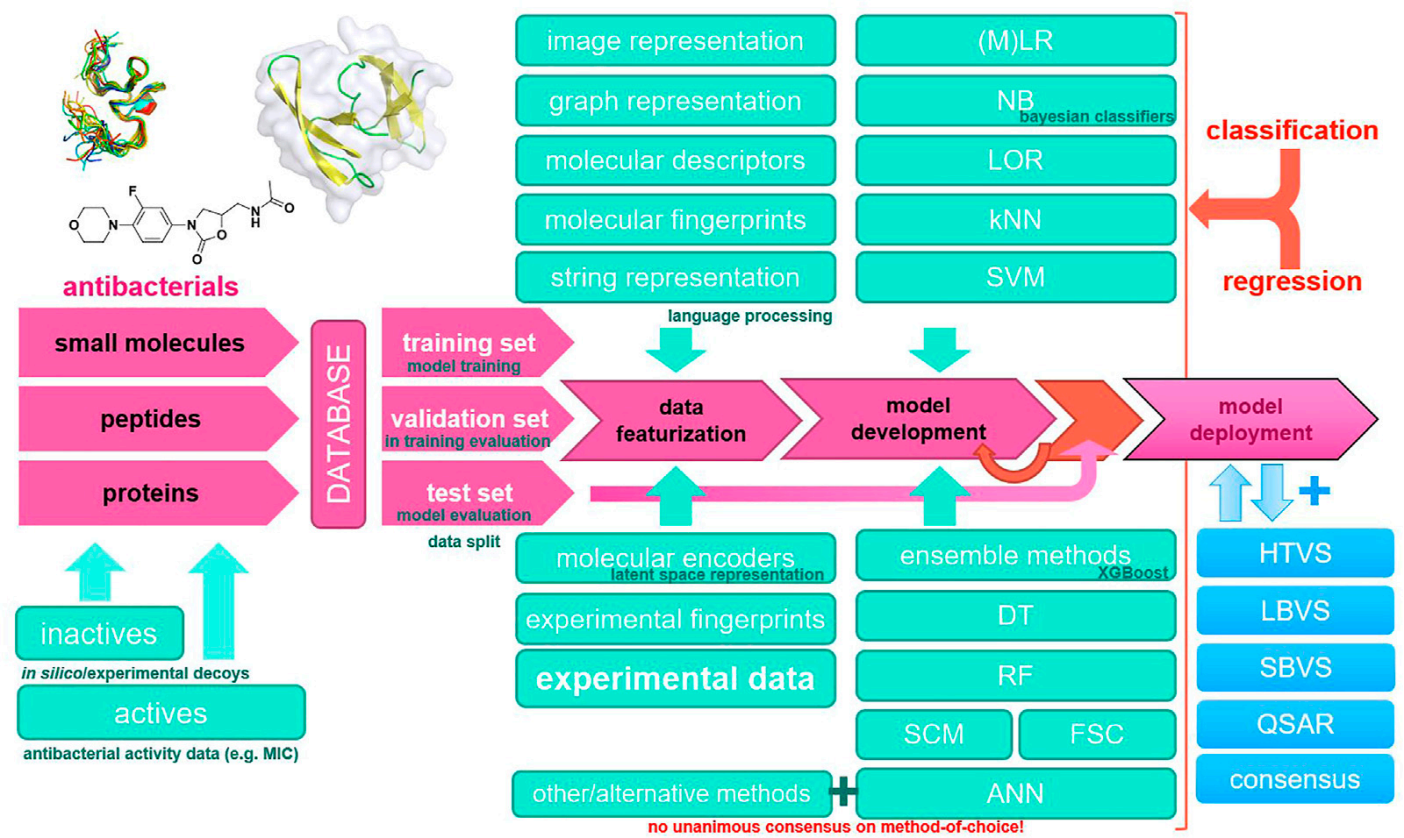
Common machine learning methodology in novel antibacterial drug design and a typical modeling workflow. ANN, artificial neural network; DT, decision tree; FSC, feedback system control; HTVS, high-throughput virtual screening; kNN, k-nearest neighbors; LBVS, ligand-based virtual screening; LOR, logistic regression; (M)LR, (multiple) linear regression; NB, naïve Bayes; QSAR, quantitative structure–activity relationship; RF, random forest; SBVS, structure-based virtual screening; SCM, set covering machine; SVM, support vector machines.

Modern antibacterial drug development currently notes a lack of novel antibacterial classes, an observation that is critical in the context of antibacterial drug resistance (Brown and Wright, 2016). Furthermore, not only single-drug resistance but also multiple-drug antibiotic resistance (MDR) has been observed in clinically relevant pathogens worldwide, rendering current established therapies ineffective (Laxminarayan et al., 2020; Vila et al., 2020). The annual number of deaths caused by infections with resistant pathogens alone is currently high and is expected to reach into millions by 2050, making high-quality data collection and reporting and antibacterial research essential (de Kraker et al., 2016; Matamoros-Recio et al., 2021). Recent advances in Computer-aided drug design (CADD) coupled with parallel and high-performance computing (HPC) platforms and new *in silico* methods represent a new paradigm for antibacterial drug discovery. In particular, machine learning methods have the potential to increase the accuracy of high-throughput virtual screening using ligand-based, structure-based, or consensus-based approaches (Serafim et al., 2020). It should be noted that modern software implementations of machine learning algorithms efficiently utilize computer hardware and are ideal for the bioinformatics or chemoinformatics scenario; however, extreme care should be taken with input data (Bzdok et al., 2017). Most importantly, the increasing availability of data makes machine learning methods even more important, either as a stand-alone method or in a consensus scenario where they can boost traditional medicinal chemistry approaches (He et al., 2021). In this review, we focus on machine learning approaches in CADD that have been reported in recent years and have been used in the development of novel antibacterials. We summarize the relevant databases and consolidate the general workflow along with the methods used in Figure 1.

## Relevant Databases for Antibacterial Drug Design

The currently accessible libraries of antibacterial compounds are enlisted that include small molecules or peptides that can be used for the design of new antibacterial agents and model development (Table 1). The reader should also be aware of tailored or focused libraries and antibacterial libraries offered by commercial compound suppliers and complete online antibacterial drug discovery communities (CO-ADD; of special mention is that the industry also contributes to the CO-ADD community, or previously SPARK-database). The ChEMBL bioinformatics platform is by far the most comprehensive resource (especially considering small molecules), followed by CO-ADD (SPARK) and antimicrobial index. Databases supporting antibacterial peptides are far more common and offer quality data.

**TABLE 1:** Currently available antibacterial compound and peptide databases suitable for *in silico* drug design^.^

Database name	Type	Location	References
ChEMBL	Comprehensive bioactivity database and bioinformatics platform	https://www.ebi.ac.uk/chembl/	Mendez et al. (2019)
Shared Platform for Antibiotic Research and Knowledge (SPARK) or CO-ADD	Community for open antimicrobial drug discovery	https://co-add.org/	Thomas et al. (2018), Cooper (2015)
Antimicrobial Index	Microorganisms and antimicrobial agents	http://antibiotics.toku-e.com/	Amirka and Qiubao, (2011)
MEGAres	Antibacterials and resistance determinants	https://megares.meglab.org/	Doster et al. (2020)
Antimicrobial Combination Networks	Antibacterial combinations	http://www.sing-group.org/antimicrobialCombination/	Jorge et al. (2016)
AntibioticDB	Antibacterial compounds	https://www.antibioticdb.com/	Farrell et al. (2018)
The Drug Repurposing Hub	Compounds, targets, and indications	https://clue.io/repurposing/	Corsello et al. (2017)
APD3	Antibacterial peptides	https://aps.unmc.edu/	Wang et al. (2016)
CAMP3	Antibacterial peptides	http://www.camp3.bicnirrh.res.in/	Waghu et al. (2016)
BAGEL4	Bacteriocins and RiPPs	http://bagel4.molgenrug.nl/	van Heel et al. (2018)
DBAASP v3	Antibacterial peptides	https://dbaasp.org/	Pirtskhalava et al. (2016)
Defensins knowledgebase	Defensins	http://defensins.bii.a-star.edu.sg/	Seebah et al. (2007)
DRAMP	Antibacterial peptides	https://ngdc.cncb.ac.cn/	Kang et al. (2019)
BaAMPs	Biofilm-active peptides	http://www.baamps.it/	Di Luca et al. (2015)
dbAMP 2.0	Antibacterial peptides	https://awi.cuhk.edu.cn/dbAMP/	Jhong et al. (2022)
AECD	Antimicrobial enzyme combinations	https://www.ceb.uminho.pt/aecd/	Jorge et al. (2019)

## Small Molecules

**FIGURE 2:**
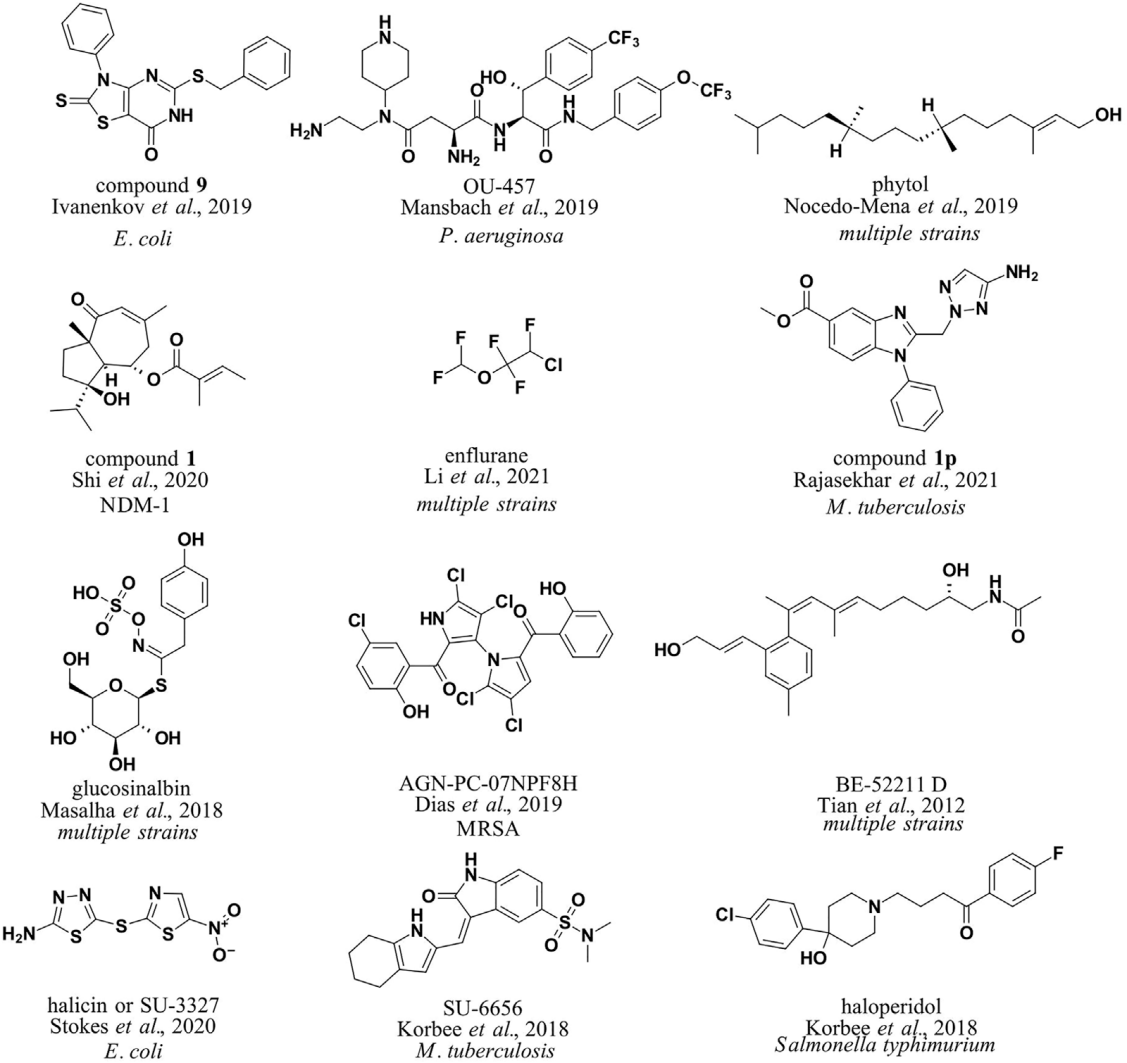
Antibacterial compounds identified by machine learning boosted *in silico* methods in CADD.

To utilize machine learning approaches in the design of antibacterial small molecules and test different machine learning approaches, Yang et al. computed a simple set of molecular descriptors for small molecules with and without antibacterial properties and evaluated the decision tree, k-nearest neighbor, and support vector machine (SVM) classification models. The authors noted the good accuracy of the SVM approach and the applicability of the methodology for antibacterial drug design. Developed models produced the best prediction accuracies of 96.66 and 98.15% for antibacterial compounds and 99.50 and 98.02% for non-antibacterial compounds (Yang et al., 2009). Ivanenkov et al. (2019) compiled a database of 145,000 small molecules, most of which came from a proprietary high-throughput screening campaign with *Escherichia coli* (*E. coli*; 1,786 active and 130,855 inactive compounds; all data points were obtained under the same experimental conditions). 1243 molecular descriptors were calculated using Dragon, ChemoSoft, MOE, and SmartMining software tools. Subsequently, self-organizing maps (Kohonen maps) were used for classification and prediction of antibacterial activity with SmartMining software, and good results were obtained (predictive power of 75.5% on average). The developed models were deployed to identify new agents against *E. coli* (compound **9**, Figure 2). Maltarollo (2019) focused on *Staphylococcus aureus* (*S. aureus*), specifically FabI inhibitors. 166 literature compounds were collected and molecular descriptors and fingerprints were calculated using PaDEL software. Decision trees (DTs), random forests (RF), multilayer perceptron (MLP), k-nearest neighbors (kNN), Naive Bayes (NB), and support vector machine (SVM) models were trained for classification. RF models performed best in classifying known connections.

Shi et al. collected a database of New Delhi metallo beta-lactamase (NDM-1) inhibitors (511 compounds) from the literature (Shi et al., 2020). This was followed by the calculation of molecular descriptors (34 descriptors, MOE software) and the representation of SMILES strings padded with zeros up to a length of 550. Different methods were tested, such as RF, SVM, and linear discriminant analysis. Finally, it was decided to use the RF model, which performed much better than the classical virtual screening model (90.5 and 69.14%, respectively). The model was used to predict potential NDM-1 inhibitors from a natural product library that contained 2,172 compounds (compound **1**, Figure 2). The authors noted that the deep-learning method was not very powerful because of low data availability. Li et al. approached in a more general manner using more data points from the ChEMBL database (Li et al., 2021). The group collected a library of 2708 active antibacterial compounds (IC_50_ cut-off of 10 μM) and 78,620 inactive compounds and proceeded to calculate fingerprints (FP2, FP3, FP4, DLFP, MACCS, ECFP2, ECFP4, ECFP6, FCFP2, FCFP4, and FCFP6) and vector representations (mol2vec, SMILES2Vec, FP2VEC software; Jaeger et al., 2018; Öztürk et al., 2018; Jeon and Kim, 2019). Several machine learning methods were reviewed, and the FP2 database along with RF, SVM, and MLP methods was selected for screening (scikit-learn library; average accuracy of 0.85). The team then constructed a predictor for antibacterial agents based on all three models and applied it to the FDA-approved small-molecule database (DrugBank, Wishart et al., 2018). Of interest is the observed low FP2 similarity (<0.2) between the predicted and FDA-approved antibacterial agents. The group focused on the nine most different predicted compounds from the FDA antibacterials with the highest screening scores in all three models; however, it did not follow up with biological evaluation. The identified compounds belonged to the classes of anticancer drugs, ocular antihypertensives, and general anesthetics, with enflurane scoring the highest. Enflurane was previously demonstrated to possess antibacterial properties *in vitro* (enflurane, Figure 2).

The superiority of machine learning–assisted molecular docking was reported by de Avila et al. (2018). The group collected a database of 22 structurally supported 3-dehydroquinate dehydratase (DHQD) inhibitors with measured inhibition constants. They developed a new polynomial scoring function with selected energy terms from classical scoring functions. Using Sandres software (Lasso and Ridge Regression), the newly developed scoring functions performed significantly better in the DHQD system test set supplemented by decoy compounds (the group did not further deploy the model).

Mansbach et al. focused on the permeation of Gram-negative bacteria and developed a fragment-based approach. They collected a database of compounds with MIC values in *Pseudomonas aeruginosa* (*P. aeruginosa*) and calculated fragment-based molecular representations for sparse regression and hierarchical clustering to identify the most relevant fragments thought to influence antibacterial activity (Mansbach et al., 2020). The method was used to predict new compounds with antibacterial properties and design “hybrid” molecules from multiple fragments (OU-457, Figure 2). Predicted molecules were experimentally evaluated.

Interestingly, an approach combining both antibacterial small molecules and antimicrobial peptides in a heterogenous library was reported by Nava Lara et al. (2019). To identify compounds with antimicrobial activity in the intestinal flora, 1444 descriptors were calculated (Padel Descriptor software) and 52 different machine learning algorithms were tested (WEKA, AutoWEKA software) to finally select a random committee algorithm classifier with receiver operating characteristic (ROC) area under the curve (AUC) performance of 0.83 for the classification. The model was applied to the FDA-approved antimicrobial agents and found that almost half of them had potential broad-spectrum activity against intestinal bacteria; however, the predictions were not experimentally substantiated. Since antibacterial peptides make up a large proportion of antibacterial chemical substances, they are discussed in more detail in the section *Antibacterial Peptides*.

Mycobacteria infections are a significant public health problem worldwide. The development of novel antimycobacterial agents remains a challenge, especially in light of the increasing emergence of multidrug-resistant strains of mycobacteria. Several reviews have been published collecting the main therapeutic targets in this field and highlighting the importance of *in silico* methods, particularly promoted by machine learning approaches and focusing on cell-wall permeability studies (Aleksandrov and Myllykallio, 2019; Pushkaran et al., 2019; Ejalonibu et al., 2021). In this way, classical approaches of virtual screening against the mycobacterial target PrpR (Vina, Glide software), MMGBSA, and molecular dynamics (MD) studies on hit compounds were complemented by the MycoCSM method to identify novel benzimidazole derivatives as potential PrpR inhibitors (compound **1p**, Figure 2; Rajasekhar et al., 2021). MycoCSM is a graph-based DT model (scikit-learn library) based on 15,000 unique compounds (featurized with RDkit descriptors) with activity against bacteria of the genus *Mycobacterium* (MIC cut-off of 1 μM), achieving correlation coefficients of up to 0.89 in predicting bioactivity in terms of minimum inhibitory concentration (Pires and Ascher, 2020).

Korbee et al. used predictive clustering trees (PCTs) to explore host-directed pathways toward antimycobacterial drug design (Clus software; https://sourceforge.net/projects/clus/). The group deployed their models on a library of pharmacologically active compounds in a (LOPAC)-based drug-repurposing screen to identify experimentally validated compounds which target receptor tyrosine kinases (RTKs) and inhibit intracellular mycobacteria (SU-6656, Figure 2) and salmonellae (haloperidol, Figure 2; Korbee et al., 2018).

## Natural Compounds

Prediction of antibacterial activity while considering molecular structure and metabolic reaction networks was also attempted by Nocedo-Mena et al. (2019) (dataset: Jeong et al., 2000). The metabolic reaction network data were merged with compounds with MIC properties in ChEMBL, and machine learning modeling with multi-output perturbations was used to build predictive models. The models were deployed to identify natural antibacterial compounds from *C. incisa* (phytol, Figure 2).

The natural compounds were further explored by Masalha et al., (2018). The group assembled a library of 628 antibacterial compounds (Comprehensive Medicinal Chemistry Database) along with an inactive set of 2892 natural compounds (AnalytiCon Discovery GmbH database) and proceeded to calculate molecular descriptors (MOE software). An iterative indexing model based on stochastic elimination was created for discriminative filtering and antibacterial identification *via* the calculated molecular bioactivity index (Rayan et al., 2010). The model ROC AUC for antibacterial classification was 0.96, and the model was deployed for screening of the natural product database to identify 10 potential antibacterial hits, two of which were experimentally confirmed as active and others are still under research (glucosinalbin, Figure 2). It is interesting to note that the authors found that comparable performance could not be achieved with either structure-based or ligand-based approaches due to non-efficient scoring or the number of false-positives.

Another report focused on marine natural sources to identify new compounds with activity against MRSA (Dias et al., 2019). Construction of a database of 6645 small molecules (ChEMBL, PubChem, ZINC; active molecules with MIC <5 μM and inactive molecules with MIC ≥5 μM) was followed by a calculation of a comprehensive list of molecular descriptors and fingerprints (PaDEL and CDK Descriptor Software) to finally build a regression model using RF, SVM, Gaussian processes (GPs), and consensus approaches for pMIC determination against MRSA. The best consensus model (*R* ^2^ of 0.68) was deployed on the StreptomeDB database and resulted in 150 hits with 12 prioritized compounds, all with confirmed anti-MRSA experimental activity (AGN-PC-07NPF8H, Figure 2). The same group also reported a nuclear magnetic resonance (^1^H and ^13^C NMR)–based approach where compounds were featurized using experimental NMR-spectra assignation data. The compound library was a dataset of 155 samples that included 50 crude extracts, 55 fractions, and 50 pure compounds obtained from microbial actinobacteria isolated from marine sediments off the Madeira archipelago. RF, SVN, and convolutional neural network (CNN) models were generated with an accuracy of 0.77 for the test set and were ready for further research and application.

Drug similarity identification was also attempted using molecular descriptors and fingerprints calculated using a database from the Current Medicinal Chemistry Database, MDL Drug Data Repot, World Drug Index (drug-like molecules), and Available Chemicals Directory for non–drug-like molecules (180,000 compounds in total). Naive Bayesian classifiers and recursive partitioning models were developed and used for drug similarity prediction in the Traditional Chinese Medicine Compound Database (TCMD) (Tian et al., 2012). The research found that the classifiers can successfully provide valuable information in the early stages of drug design (drug-like compound identification accuracy of 0.86) and identify important drug-like scaffolds and even classify them by pharmacological activity, for example, label scaffolds of antibacterial compounds (BE-52211D, Figure 2).

Indeed, natural compounds represent an invaluable source of chemical diversity, and their drawbacks (availability, complexity, synergistic pharmacodynamics) in drug development could be mitigated by modern machine learning methods (Rodrigues et al., 2016). To this end, Zhang et al. have collected several machine learning protocols for activity prediction of natural products (Zhang et al., 2021).

## Antibacterial Peptides

An important subfield of the discovery of new antibacterials is also the discovery of antibacterial peptides. The latter can serve as active agents, starting points for the design of peptidomimetics, or probes for further studies. The field and *in silico* tools have been reviewed previously (Lee et al., 2017; Cardoso et al., 2020; Wang et al., 2021), with the emphasis on machine learning–enabled antimicrobial peptide discovery and SVM for the discovery of membrane-active peptides (Lee et al., 2018). However, Frecer reported a successful design of cationic antibacterial peptides derived from protegrin-1 as early as 2006 (Frecer, 2006), and machine learning methodology contributed significantly to the design and discovery of novel peptides, as demonstrated by Fjell et al. To single out just one report, they reinforced the traditional QSAR approach with an artificial neural network model (ANN) that inferred a set of peptides with known antibacterial properties from computed descriptors (MOE software). After deploying the model in a screening scenario (*in silico* library with random peptides), short cationic peptides with MICs in the range of 0.3–10 μM were identified (Fjell et al., 2009). The extended research group later reported an interesting approach for relational learning algorithms (RelF and WEKA software for regression) to explore patterns from the relational structures of the antibacterial peptides or an approximate attribute-value representation of the peptides (Szaboova et al., 2012). Feature vectors for peptide representation were also usedusing Chou’s pseudo-amino acid composition (PseAAC), and the SVM was successfully used to classify antibacterial peptides (Khosravian et al., 2013).

The later approaches were also extended beyond antibacterial peptide identification to peptide target selectivity or prediction of Gram-positive or Gram-negative activities (Veltri et al., 2015). The group used an evolutionary feature construction and a fast correlation-based filter selection algorithm with logistic regression (WEKA) to successfully identify antibacterial peptides of up to 11 amino acids in length. The same group used APD3 database, converted peptide sequences into zero-padded numerical vectors of length 200, and trained a deep neural network (DNN; Keras, TensorFlow software) model to classify antimicrobial peptides (accuracy of 0.98 on APD3 data). Embedding vector visualization was also performed, and a reduced alphabet learnt from the DNN model was developed. Reduced sequence space retained good classification performance (Veltri et al., 2018). Müller et al. trained a recurrent neural network (RNN) with helical antimicrobial peptides (1554 peptides, APD). The sequences were padded according to the length of the longest sequence, N-terminal token added, and One-hot encoding employed (Müller et al., 2018). The resulting model was developed for *de novo* sequence generation, where 82% were predicted to be active antimicrobial peptides compared to 65% of randomly sampled sequences with the same amino acid distribution as the training set (CAMP AMP prediction tool; Waghu et al., 2014). Wu et al. used previous amino acid substitution data for antibacterial peptides and developed an amino acid activity contribution matrix (Wu et al., 2014). Using this methodology, the group developed a 12-mer DP7 peptide with antibacterial properties against multiple strains (Zhang et al., 2019). Similarly, Yoshida et al. used a natural antibacterial peptide Temporin-Ali (FFPIVGKLLSGLL-NH2) and PSI BLAST to create a library of distantly related and functionally similar sequences, prepared the peptides, and evaluated their antibacterial activities *in vitro* on *E. coli* to construct a fitness matrix. The data were then used to train a model and deploy it to optimize peptide sequences. The group produced a peptide with 163-fold lower activity on *E. coli* bacteria (Yoshida et al., 2018). Another approach using rough set theory constructed quantitative structure–activity relationship rules for existing antibacterial peptides. New sequence development *via* a genetic algorithm and further *in vitro* testing resulted in a peptide being active against *Staphylococcus epidermidis* (*S. epidermidis*) (Boone et al., 2021).

Approaches were again extended by considering toxicity data in the development of novel antibacterial peptides intended for human drug development campaigns. Capecchi et al. used the Database of Antimicrobial Activity and Structure of Peptides (DBAASP; 4774 active peptides with an MIC threshold of 32 mg/ml) to train a recurrent neural network (RNN) generative model to develop nonhemolytic antibacterial peptides with activity against *P. aeruginosa*, *Acinetobacter baumannii* (*A. baumannii*), MRSA, and a broader range of MDR strains. To test the performance of machine learning models for antibacterial peptide design, Wani et al. trained models on a database of antibacterials (2638) and inactive peptides (3700) using RF, kNN, SVM, DT, NB, quadratic discriminant analysis (QDA), and ensemble learning. RF models were found to perform best in validation experiments. The group also highlighted three important peptide descriptors as essential for antibacterial activity, namely, charge, polarity, and pseudo-amino acid composition (Wani et al., 2021). The field of *in silico* tools for designing antibacterial peptides using machine learning is also gaining traction, and targeted tools such as AMPGAN v2 are being developed (Van Oort et al., 2021). AMPGAN v2 is a bidirectional conditional generative adversarial network (BiCGAN) that targets *de novo* generation of antibacterial peptides. The group used training data by compiling the Database of Antimicrobial Activity and Structure of Peptides (DBAASP), Antiviral Peptide database (AVPdb), and UniProt databases (Apweiler et al., 2004; Gogoladze et al., 2014; Qureshi et al., 2014).

## Antibacterial Drug Resistance

Machine learning approaches are also being used to combat antibiotic resistance. Back in 2017, Macesic et al. published a review of antibacterial susceptibility testing using genotype–phenotype prediction, machine learning approaches to identify resistant strains, and the use of machine learning to improve treatment and optimize clinical approaches to MDR infections (Macesic et al., 2017). Interestingly, the authors lamented data abstraction and quality but pointed out that the methodology gains strength with the availability of quality data. A recent review article discusses several bioinformatics approaches involving machine learning that are useful for studying bacterial resistance, such as the use of modern bioinformatics approaches for the interpretation of data from increasing sequencing libraries; study of protein structures; *in silico* analysis of serovar, serogroup, and antigen markers; the development of *in silico* plasmid detection methods; *in silico* identification of resistance genes; antibacterial surveillance; and in turn, the prediction of the evolution of antibacterial drug resistance (Ndagi et al., 2020). In addition, machine learning approaches have been used beyond resistance prediction using genomic data to elucidate resistance mechanisms and for antibacterial stewardship applications. The latter are mainly concerned with patient data analysis, diagnosis, treatment, and prevention of resistance development in a clinical scenario (Anahtar et al., 2021). With the increasing use of antibiotics and the accompanying bacterial resistance, we cannot overemphasize the importance of these new approaches in translational research. Furthermore, the power of reported methods is increasing with the growth of quality data and availability of curated and resistance-focused libraries such as Plasmid ATLAS by Jesus et al., (2019), Ensembl Genomes (Bacteria) by Yates et al., (2022), BacDive by Reimer et al., (2019), Virulence Factor Database VFDB by Chen et al., (2005), Beta-Lactamase Database (BLDB) by Naas et al., (2017), Antibiotic Resistance Genes Database (ARDB, Liu and Pop, 2009), BacMed (Pal et al., 2014), and Comprehensive Antibiotic Resistance Database (CARD, McArthur et al., 2013 and Alcock et al., 2020).

## Modern Approaches

As reviewed already by Durrant and Amaro in 2014 (Durrant and Amaro, 2015) up to now, the medicinal chemistry community and pharmaceutical industry are adopting machine learning techniques in medicinal chemistry and drug design in general (Ekins et al., 2019) and antibacterial drug development (Patel et al., 2020; Serafim et al., 2020). Of special mention would be the acknowledgment of enormous data availability, its application toward drug design (Burki, 2020), and utilization of modern artificial intelligence approaches (David et al., 2021). Specifically, the applications of modern deep learning methods in antibacterial drug design are evident from a multitude of published reports in scientific literature, tailored offerings by commercial drug design software developers, and emergence of deep-learning in drug design–focused CROs and start-ups (Schroedl, 2019; Chang et al., 2019; Gupta et al., 2021; da Silva et al., 2021).

### Deep-Learning and Artificial Neural Networks

An excellent example of the development and use of deep learning supervised, semi-supervised, or unsupervised models in the area of novel antibacterial drug development and discovery was recently reported (Stokes et al., 2020). The group initially generated the dataset by computing graph representations, Morgan fingerprints, and molecular features computed using RDKit (internal training set of 2560 compounds, 120 positive controls; with a test set: Broad’s Drug Repurposing Hub of 6111 compounds) and used a Directed Message Passing Neural Network (D-MPNN; Chemprop implementation available on Github), a type of graph convolutional neural network for model development. After prioritization by toxicity prediction, the authors identified one promising new antibiotic, halicin (SU -3327, Figure 2), and eight (ZINC000098210492, ZINC000001735150, ZINC000225434673, ZINC000019771150, ZINC000004481415, ZINC000004623615, ZINC000238901709, and ZINC000100032716) other potential antibiotic candidates and experimentally validated the obtained hits to have an antibiotic activity on *E. coli*.

### K-Nearest Neighbor

kNN is a supervised learning method that can be applied for classification and regression tasks and is effectively utilized in medicinal chemistry for novel antibacterial drug design. A classification application of kNN was reported by Karakoc et al. for classification of small molecules based on selecting the most relevant set of chemical descriptors used for ultimate discrimination between active and inactive compounds on various biological systems (Karakoc et al., 2007). A comprehensive list of kNN applications in classification and regression tasks all applied toward drug delivery for infectious disease treatment, treatment regimen optimization, drug delivery system and administration route design, and drug delivery outcome prediction was reported by He et al. (2021).

### Support Vector Machines

SVM supervised learning models are also widely applied for classification, regression, and ranking/virtual screening tasks in medicinal chemistry in a range of fields such as novel anticancer research, design of antivirals, protein–protein interaction research etc. (Romero-Molina et al., 2019). Focusing on antibacterial drug design, Li et al. reported SVM model development from the fingerprint-featurized ChEMBL database in order to identify novel antibacterial compounds (Li et al., 2021). SVM model applications in antibacterial design and antibacterial drug resistance research were reviewed by Serafim et al. (2020). In a broader scope, recent advances in SVMs and their numerous drug discovery applications are summarized by Maltarollo et al. (2019).

### Random Forest and Decision Trees

RF is a supervised ensemble learning method that consists of a multitude of decision trees, constructed at a training phase. Upon reviewing literature on novel antibacterial design supported by machine learning, RF models were found to be one of the most commonly applied for classification, regression, and other tasks and represent a performance and computationally lean approach. In this review, a number of RF applications are presented, for small molecules, peptides (Bhadra et al., 2018), natural product–based antibacterial design, and studying antibacterial drug resistance (Dias et al., 2019; Maltarollo et al., 2019; Shi et al., 2020; Li et al., 2021; Wani et al., 2021). A good example of underlying supervised learning DT method was reported by Suay-Garcia et al. (2020). The authors created a QSAR model to predict antibacterial activity against *E. coli*. The compounds were classified using a tree-based method and linear discriminant analysis. A comprehensive review on other DT applications is also provided by Serafim et al., (2020).

### Coupling to Big Data

Needless to say, we must emphasize the coupling of modern machine learning approaches to valuable data sources. Sripriya Akondi et al., (2022) emphasize the use of compound and protein conformational data which in its abundance classifies as big data in all respects. However, common problems with big data sources such as data quality, over-fitting, and difficult or lengthy protocols should be taken in consideration (Motamedi et al., 2022). Taken together, the big data era will walk hand-in-hand with future drug design and will have a significant impact on how to approach a drug discovery campaign (Zhu, 2020; Bhattarai, et al., 2022; Lee et al., 2022). Zhao et al. point out in a wonderful report “10 Vs.” or characteristics that are intrinsic in drug discovery big data that we should be aware of and utilize, namely: volume (size of data), velocity (data growth), variety (lots of data sources), veracity (variable data quality), validity (authenticity of data), vocabulary (aware of the terminology), venue (numerous data platforms), visualization (presentation and patterns in data), volatility (time domain of the data and usefulness time window), and value (associated economic and added value, Zhao et al., 2020).

## Conclusion

In conjunction with antibacterial compound databases (Table 1) and general (big) data sources such as ChEMBL and CO-ADD (SPARK), efficient research in the area of new antibacterial drug design and target identification is possible (Gaulton et al., 2017; Wishart et al., 2018). Incorporating novel machine learning methods can successfully boost the traditional medicinal chemistry approaches, and this review highlights a host of applications and machine learning model deployments. The examples include synthetic and natural small molecules, as well as peptides, ranging from a narrow spectrum of Gram-positive or Gram-negative bacteria to a broad spectrum of compounds acting on mycobacteria and eventually even MDR bacteria. However, in reviewing the literature, it is immediately apparent that medicinal chemistry is currently still in the introductory phase of exploring modern (and also established) machine learning methods and adapting them to the field. Most of the reports are proof-of-concept works where the models are only deployed to test the data and no experimental biological evaluation is performed. However, the analysis of the best performing featurization approaches and the methods themselves may be even more important takeaways.

Input data is of critical importance, and the available tailored or focused antibacterial data libraries, especially public resources, leave much to be desired. The good availability of antimicrobial peptide data and general relational databases, such as the ones mentioned above, improves the situation. In conclusion, the immense value of modern machine learning methods is obvious—coupled with classical and experimental approaches in medicinal chemistry— and new advances in antibacterial drug design and mode of action research are possible.

## Abbreviations

AI, artificial intelligence; ANN, artificial neural network; CADD, computer-assisted drug design; DT, decision tree; FSC, feedback system control; kNN, k-nearest neighbors; LOR, logistic regression; (M)LR, (multiple) linear regression; MDR, multidrug resistant; MIC, minimum inhibitory concentration; MRSA, methicillin-resistant *Staphylococcus aureus*; NB, naïve Bayes; RF, random forest; RiPPS, ribosomally synthesized and posttranslationally modified peptides; SCM, set covering machine; SVM, support vector machines.
